# Molecular Effects of Low-Intensity Shock Wave Therapy on L6 Dorsal Root Ganglion/Spinal Cord and Blood Oxygenation Level-Dependent (BOLD) Functional Magnetic Resonance Imaging (fMRI) Changes in Capsaicin-Induced Prostatitis Rat Models

**DOI:** 10.3390/ijms23094716

**Published:** 2022-04-25

**Authors:** Hung-Jen Wang, Chia-Hao Su, Yu-Ming Chen, Chun-Chieh Yu, Yao-Chi Chuang

**Affiliations:** 1 Department of Urology, Kaohsiung Chang Gung Memorial Hospital and Chang Gung University College of Medicine, Kaohsiung 833, Taiwan; hujewang@gmail.com (H.-J.W.); corn88504047@gmail.com (Y.-M.C.); 2 Center for Shockwave Medicine and Tissue Engineering, Kaohsiung Chang Gung Memorial Hospital and Chang Gung University College of Medicine, Kaohsiung 833, Taiwan; 3 Institute for Translational Research in Biomedicine, Kaohsiung Chang Gung Memorial Hospital, Kaohsiung 833, Taiwan; chiralsu@gmail.com (C.-H.S.); knightaws0@gmail.com (C.-C.Y.)

**Keywords:** pain, shock wave, fMRI, dorsal root ganglion, NGF, BDNF, TRPV1, peripheral nervous system

## Abstract

Neurogenic inflammation and central sensitization play a role in chronic prostatitis/chronic pelvic pain syndrome. We explore the molecular effects of low-intensity shock wave therapy (Li-ESWT) on central sensitization in a capsaicin-induced prostatitis rat model. Male Sprague–Dawley rats underwent intraprostatic capsaicin (10 mM, 0.1 cm^3^) injections. After injection, the prostate received Li-ESWT twice, one day apart. The L6 dorsal root ganglion (DRG)/spinal cord was harvested for histology and Western blotting on days 3 and 7. The brain blood oxygenation level-dependent (BOLD) functional images were evaluated using 9.4 T fMRI before the Li-ESWT and one day after. Intraprostatic capsaicin injection induced increased NGF-, BDNF-, and COX-2-positive neurons in the L6 DRG and increased COX-2, NGF, BDNF, receptor Trk-A, and TRPV1 protein expression in the L6 DRG and the dorsal horn of the L6 spinal cord, whose effects were significantly downregulated after Li-ESWT on the prostate. Intraprostatic capsaicin injection increased activity of BOLD fMRI responses in brain regions associated with pain-related responses, such as the caudate putamen, periaqueductal gray, and thalamus, whose BOLD signals were reduced after Li-ESWT. These findings suggest a potential mechanism of Li-ESWT on modulation of peripheral and central sensitization for treating CP/CPPS.

## 1. Introduction

Chronic prostatitis/chronic pelvic pain syndrome (CP/CPPS) is a common disease affecting the quality of life in 5–10% of adult males in North America [1]. The symptoms of CP/CPPS include pelvic pain (prostate, perineum, or urethra pain) and various urinary dysfunctions [2]. Although the exact mechanisms of CP/CPPS have not been completely established, the possible causes of CP/CPPS have been linked to the interaction between immune, endocrine, neurological, and psychological factors, all attributed to pelvic pain and related symptoms [3]. The characteristic of chronic pain in CP/CPPS has been demonstrated to relate to neurogenic inflammation, which describes the activation of prostate afferent nerves by prostate inflammation and possible sensitization of the central nervous system [3].

Low-intensity extracorporeal shock wave therapy (Li-ESWT) has been applied as a noninvasive therapeutic method for urological diseases [4]. For treating CP/CPPS, Li-ESWT has been clinically applied since 2009 [5]. Several randomized, placebo-controlled studies have confirmed that Li-ESWT significantly improved pain, quality of life, and voiding dysfunction compared with placebo treatment [5,6,7] in CP/CPPS patients. The effects of Li-ESWT on the inhibition of pain have been suggested by nociceptor hyperstimulation followed by hyposensitivity and interruption of pain nerve impulses [8]. However, the effects of Li-ESWT on remodeling the neuroplasticity of pain memory and resetting the pain pathway have not been studied.

Previous studies in rats have shown that intraprostatic capsaicin injection stimulated C-afferent fibers, upregulated expression of cyclooxygenase-2 (COX-2), nerve growth factor (NGF), and other inflammatory mediators in the prostate, and induced pain behaviors [9], which were suppressed by Li-ESWT in a time- and dose-dependent fashion. However, the effects of Li-ESWT on modulation of central sensitization and pain expression in functional brain areas, which play a role in chronic pain, are still unclear. To advance our previous findings of Li-ESWT effects on inhibition of inflammatory molecular on peripheral organs and pain behaviors, we explore the effects of Li-ESWT on neuromodulation by evaluating the L6 dorsal root ganglion (DRG)/spinal cord and functional magnetic resonance imaging (fMRI) blood oxygenation level-dependent (BOLD) signal changes in brain regions related to pain in capsaicin-induced prostatitis rat models.

## 2. Results

### 2.1. Immunostaining—Li-ESWT Decreased L6 DRG COX-2-, NGF-, and BDNF-Positive Neurons Induced by Intraprostatic Capsaicin Injection

**Figure 1:**
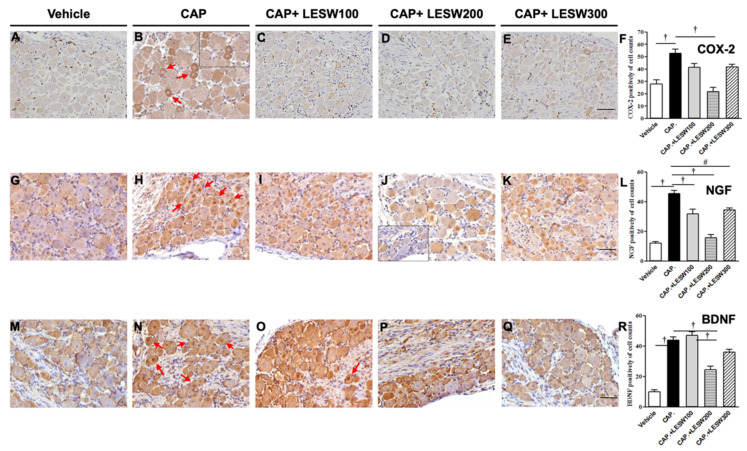
Immunohistochemistry staining of L6 DRG on day three post capsaicin injection with or without Li-ESWT. Intraprostatic capsaicin (CAP) injection-induced increase in COX-2, NGF, and BDNF expression in the L6 DRG (**B**,**H**,**N**, red arrow), which effects were decreased in the 200 and 300 shock wave treatment groups (**D**,**E**). All three groups of Li-ESWT significantly decreased NGF immunoreactivity (**L**), while only 200 shocks of Li-ESWT significantly decreased COX-2 and BDNF-positive neurons (**F**,**R**). (**A**–**E**): COX-2 stain; (**G**–**K**): NGF stain; (**M**–**Q**): BDNF stain; (**F**,**L**,**R**): positive neurons per visual field. Magnification ×400; inlet, magnification ×400; scale bars indicate 50 μm. Statistically significant difference between groups (*n* = 10 per group; # *p* < 0.01; † *p* < 0.001) (**F**,**L**,**R**).

Three days after intraprostatic capsaicin injection, immunoreactivity of L6 DRG neurons for COX-2, NGF, and BDNF was significantly higher than that of vehicle injection (Figure 1; the relative percentage was 188.4%, 372.1%, and 433.7%, respectively). Most of the COX-2-, NGF-, and BDNF-positive neurons are small (<20 μm in diameter) and medium (20–30 μm in diameter) sized. LESW decreased the expression of COX-2-positive neurons on day 3 in the 100, 200, and 300 shock wave groups (Figure 1A–E; 21.3%, 58.7%, * and 20.9% reductions, respectively; * *p* = 0.001; Figure 1F). Similar effects were observed for NGF- and BDNF-positive neurons (Figure 1G–Q; NGF (+) neurons, 30.3%, 65.2%, and 24.5% decreased compared with those in the capsaicin group *p* = 0.001, 0.001, and 0.01, respectively, Figure 1L; BDNF (+) neurons, 7.3%, 44.1%,* and 20.1% changed compared with those in the capsaicin group; * *p* = 0.001, Figure 1R).

### 2.2. Immunostaining—Li-ESWT Decreased Upregulation of Trk-A and TRPV1 Immunoreactivity in L6 DRG Induced by Intraprostatic Capsaicin Injection

**Figure 2:**
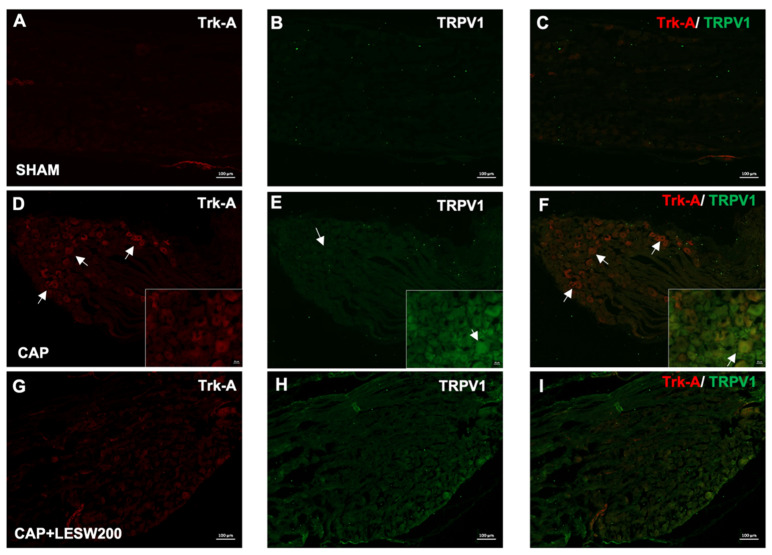
Co-localization of Trk-A and TRPV1 immunofluorescence staining in L6 DRG on day three after intraprostatic capsaicin (CAP) injection. Double immunostaining showed that Trk-A immunoreactivity (red staining) was co-localized with TRPV1(green staining). Most of the TRPV1 positive neurons expressed Trk-A (white arrow). intraprostatic capsaicin (CAP) injection-induced increment of TRPV1 and Trk-A immunoreactivity in L6 DRG, and this effect was ameliorated by 200 shocks of Li-ESWT. (**A**–**C**) after vehicle injection; (**D**–**F**) after intraprostatic capsaicin (CAP) injection. (**G**–**I**) intraprostatic capsaicin injection with 200 shock wave treatment. Scale bars indicate 100 μm.

**Figure 3:**
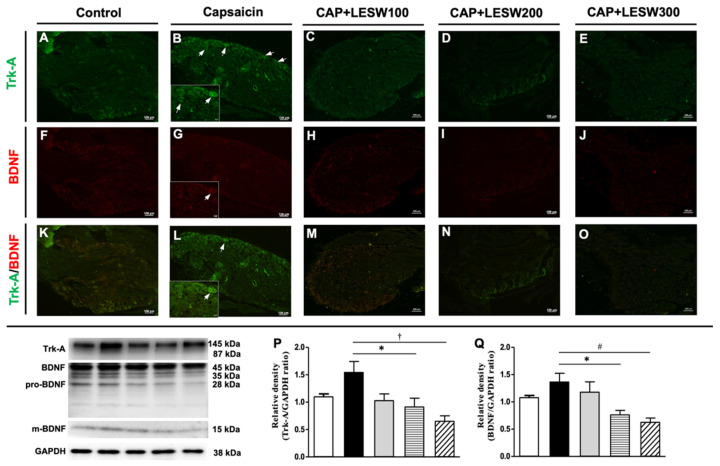
Immunofluorescence staining and Western blots analysis of L6 DRG three days after intraprostatic capsaicin (CAP) injection. Double immunostaining showed that Trk-A immunoreactivity (green staining) was co-localized with BDNF (red staining). Most of the BDNF-positive neurons expressed Trk-A (white arrows). Trk-A and BDNF immunoreactivity increased in intraprostatic capsaicin (CAP) injection group (**D**,**E**,**F**), which effects were attenuated by 200 and 300 shock wave treatment (**L**,**O**). Western blots for detecting Trk-A and BDNF expression in DRG showed consistent results with immunofluorescence staining. In total, 200 and 300 shocks of Li-ESWT decreased overexpression of the Trk-A and BDNF induced by intraprostatic capsaicin injection (**P**,**Q**). (**A**–**E**): Trk-A stain; (**F**–**J**): BDNF stain; (**K**–**O**): Trk-A and BDNF double stain; magnification ×400; inlet, magnification ×400; scale bars indicate 100 μm. Statistically significant difference between groups (*n* = 10 per group; * *p* < 0.05; # *p* < 0.01; † *p* < 0.001) (**P**,**Q**).

The Trk-A- and TRPV1-positive neurons in the L6 DRG increased on day 3 after intraprostatic capsaicin injection. Constitutively expressed Trk-A colocalizes with TRPV1, and the increased immunoreactivity was suppressed by a 200-shock wave treatment (Figure 2). Double immunostaining showed that Trk-A immunoreactivity was co-localized with BDNF. BDNF-positive neurons associated with the expression of Trk-A immunoreactivity were increased in the prostatitis groups (Figure 3B,G,L), whose effects were attenuated by 200 and 300 shocks of Li-ESWT (Figure 3N,O).

### 2.3. Western Blotting—Li-ESWT Suppressed Upregulation of COX-2, NGF, and BDNF and Trk-A and TRPV1 in L6 DRG/Spinal Cord Induced by Intraprostatic Capsaicin Injection

**Figure 4:**
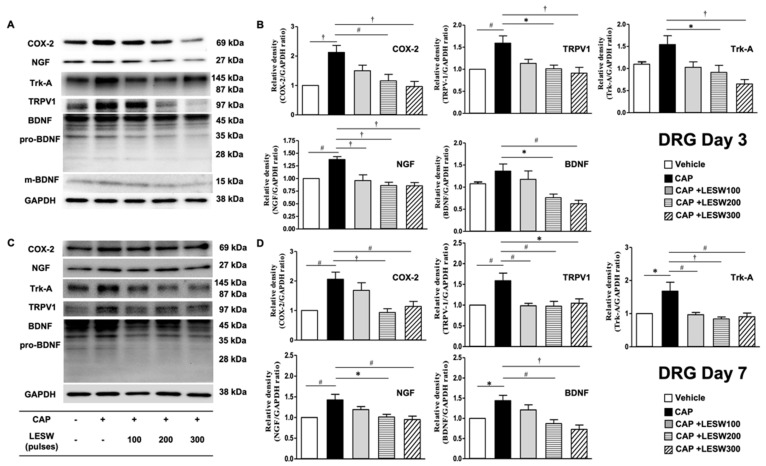
(**A**,**C**) Western blot for detecting COX-2, NGF, Trk-A, BDNF, and TRPV1 expression in the L6 DRG on day three and day seven after intraprostatic capsaicin injection. (**B**,**D**) All inflammatory molecules, neurogenic growth factors and receptors were upregulation (solid black bar) on day three and day seven. Li-ESWT significantly suppressed COX-2, NGF, Trk-A, BDNF, and TRPV1 expression in a dose-dependent manner. (*n* = 10 per group; * *p* < 0.05; # *p* < 0.01; † *p* < 0.001).

A Western blot of L6 DRG demonstrated that intraprostatic capsaicin injection induced a significant increase in the expression of COX-2, NGF, and BDNF and Trk-A and TRPV1 on day 3 compared to that in the sham (vehicle) group (Figure 4, Table 1), whose effects were suppressed by Li-ESWT. (Figure 4B; *p* < 0.05 in the 200 and 300 shock wave treatment groups). The impact of LESW treatment can last for 1 week (Figure 4D). The results of Western blotting of Trk-A and BDNF expression in L6 DRG were consistent with the results of immunofluorescence staining (Figure 3P,Q and Figure 4B).

**Figure 5:**
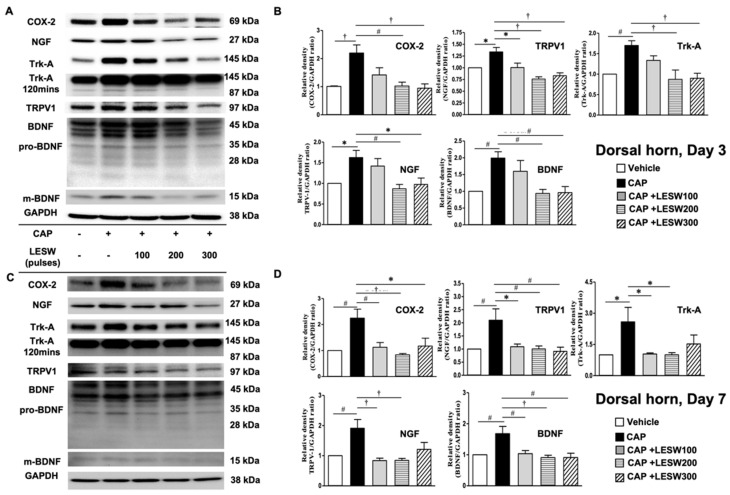
(**A**,**C**) Western blot for COX-2, NGF, Trk-A, BDNF, and TRPV1 expression in the dorsal horn of L6 spinal cord on day 3 and day 7 after intraprostatic capsaicin injection. (**B**,**D**) Capsaicin injection increased COX-2, NGF, Trk-A, BDNF, and TRPV1 expression (solid black bar) on day three and day seven in the L6 spinal cord. In total, 200 and 300 shocks of Li-ESWT significantly suppressed expression of inflammatory molecules, neurogenic growth factors and receptor expression on day three after treatment. By day seven, all shock wave treatment groups showed significant downregulation of the COX-2, TRPV1 and BDNF expression, while only 100 and 200 shocks of Li-ESWT significantly decreased NGF and Trk-A expression. (*n* = 10 per group; * *p* < 0.05; # *p* < 0.01; † *p* < 0.001).

The effects of intraprostatic capsaicin injection and Li-ESWT on the L6 dorsal horn are similar to the effects on the L6 DRG. The expression of COX-2, NGF, BDNF, Trk-A, and TRPV1 was upregulated (Figure 5, Table 2) after intraprostatic capsaicin injection. Two hundred and 300 shocks of Li-ESWT on the prostate significantly suppressed these inflammatory molecules, neurogenic growth factors, and receptor expression on day 3 (Figure 5B). By day 7, all shock wave treatment groups showed the ability to significantly downregulate the COX-2, TRPV1, and BDNF expression, while only 100 and 200 shocks of Li-ESWT significantly decreased NGF and Trk-A expression (Figure 5D).

### 2.4. Li-ESWT Suppressed the Progression of BOLD fMRI Signal Changes That Increased with Time in Prostatitis Rats

**Figure 6:**
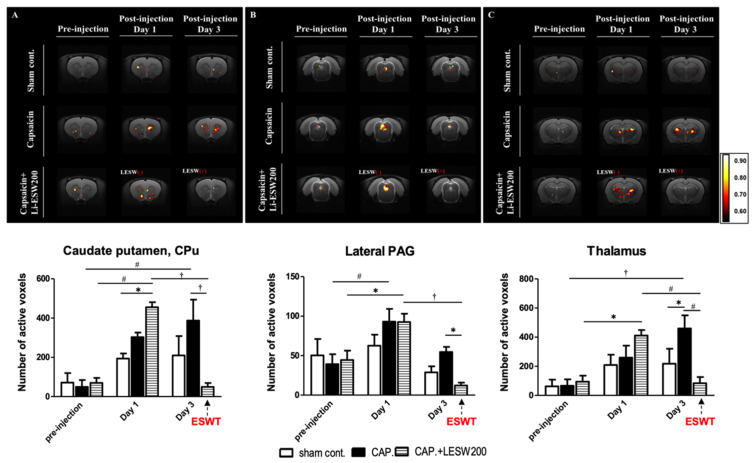
BOLD fMRI signal changes over time with or without Li-ESWT in capsaicin-induced prostatitis rats. Shown here are activation maps of BOLD signal changes in ROI includes (**A**)caudate putamen; (**B**)lateral PAG and (**C**)thalamus. One day after capsaicin injection, positive BOLD signal changes increased in caudate putamen, lateral PAG, and thalamus. In Li-ESWT treated rats, the number of active voxels in these three pain-related ROI was significantly decreased by day three. However, in rats without having Li-ESWT, the active voxels in caudate putamen and thalamus continue to increase by day 3. (*n* = 8 per group; * *p* < 0.05; # *p* < 0.01; † *p* < 0.001).

We analyzed the BOLD signal changes in pain-related ROIs, including the caudate–putamen, lateral PAG, and thalamus. On day 1 after capsaicin injection, positive BOLD signal changes increased in the caudate—putamen, lateral PAG, and thalamus by 615.3%, 237.4%, and 392.9%, respectively (Figure 6). In Li-ESWT-treated rats, the number of active voxels in these three pain-related ROIs was significantly decreased by day 3 compared with that of the CAP group (83.5 ± 43. vs. 459.8 ± 9.0; 12.2 ± 3.7 vs. 54.5 ± 6.5; 49 ± 20.2 vs. 387.4 ± 106.4 in the Th, LPAG, and CPu, with *p* < 0.01, *p* < 0.05, and *p* < 0.001, respectively). However, in rats without Li-ESWT, the active voxels in the caudate–putamen and thalamus continued to increase by day 3 (Figure 6, Table 3).

## 3. Discussion

Neurogenic inflammation has been suggested to be one of the crucial causes of CP/CPPS, which activates the peripheral and central nervous systems. Previous studies have provided data for major remodeling, as evidenced by increased c-fos expression at L6 and S1 in chemical-induced cystitis or prostatitis models in rats [10,11]. Furthermore, Ishigooka et al., have shown significant overlaps of nociceptive neurons within the spinal cord, which receives nociceptive inputs from the pelvic soma and viscera [10]. The nociceptive effect might lead to persistent pain even if the inflammatory condition has subsided. The consensus is that the sensitization of the central nervous system (CNS) plays an essential role in the development and maintenance of chronic pain. Previous literature provides evidence for the presence of central sensitization in urogynecological chronic pelvic pain with changes in brain morphology/function and sensory function [12].

NGF has been known to play an essential role in the development of chronic pain. NGF is secreted by epithelial cells and immune cells such as mast cells in the inflamed area. NGF activates Trk-A receptors on sensory neurons, and the neurotrophin-receptor complex endosome transmits NGF signal from the targeted nociceptive neuron, which further activates Rap1/Erk1/2, p38 MAPK, and PI3K/Akt pathways that lead to enhanced peripheral sensitization [13]. Miller et al. have demonstrated that the NGF level detected from the seminal plasma or expressed prostatic secretions of CP/CPPS patients correlated with pain and symptom severities, whose level was normalized after successful treatment [14]. It has been well established that inflamed tissues significantly increase the NGF level, which might be secondary to inflammatory cytokines i.e., tumor necrosis factor-α (TNF-α), interleukin-1β (IL-1β), transforming growth factor (TGF)- α and TGF-β1 [15,16,17]. Li-ESWT exerts anti-inflammatory effects through the PI3K/AKT/FOXO1 signaling pathway in autoimmune prostatitis rat models [18]. Additionally, mast cells synthesize and release biologically active NGF in neuroimmune interactions [19], which was suppressed by Li-ESWT in a prostatitis rat model induced by intraprostatic injection of 1% carrageenan [20]. Additionally, Li-ESWT has been demonstrated to suppress HCl-induced bladder overactivity in association with a decrease in the inflammatory reaction and mast cell infiltration [21]. We suggest that Li-ESWT has effects on inhibition of mast cell activity and suppression of NGF released in the inflamed prostate. Our previous studies revealed that intraprostatic capsaicin injection activated C-afferents and induced pain behavior and inflammatory reactions, including increased inflammatory cell accumulation, COX-2 expression, and plasma extravasation [9,22,23]. Li-ESWT affects the inhibition of NAcht, leucine-rich repeat, and PYD domain-containing protein 1 (NALP1), caspase1, IL-1β, COX-2, TNF-α, and NGF expression and reduces prostatic pain and inflammation [9]. Furthermore, our current study provided evidence of the upregulation of NGF and Trk-A expression in the L6 DRG/spinal cord after intraprostatic capsaicin injection, whose effects were ameliorated through prostate-targeting Li-ESWT. The protein levels of TRPV1 and BDNF in the L6 DRG/spinal cord were increased in capsaicin-induced prostatitis rats and were downregulated by Li-ESWT.

The abundant TRPV1 sensory innervation was found in the human prostate with CP/CPPS. TRPV1-immunoreactive nerve fibers are present in the lamina propria of the prostatic stroma [24]. Intraprostatic injection of capsaicin evoked TRPV1-expressing afferent fibers and transmitted sensory signals to the CNS, which generated a painful sensation. The TRPV1-containing nerve also exerts efferent functions by releasing a cocktail of neuropeptides, such as calcitonin gene-related peptide, neurokinin A, and substance P. These neuropeptides further induce neurogenic inflammation and induce the excitability of afferent nerves [25]. Furthermore, inflammation enhanced NGF secretion, activated second messenger signaling cascades, and caused a direct sensitization of TRPV1 [26]. In an experimental autoimmune prostatitis rat model, TRPV1 expression was increased in the prostate and L5–S1 DRG neurons with upregulation of NGF and Trk-A [27,28]. These findings are consistent with our results that intraprostatic capsaicin injection increased TRPV1 protein expression in the dorsal root ganglia and spinal cords. These increments of TRPV1 activity were reduced by Li-ESWT, especially in the 200 and 300 shock wave groups on days 3 and 7 after capsaicin injection. We proposed that Li-ESWT might decrease the secretion of NGF in inflamed tissues or the downregulation of PI3K signal transmission pathways and reduce the expression of TRPV1 [18,29].

Furthermore, NGF might also upregulate BDNF in Trk-A-positive DRG neurons [30,31]. Intraprostatic capsaicin injection might induce NGF increment in the prostate and enhance Trk-A and BDNF upregulation in the L6 DRG. We found that BDNF is expressed mainly in a subpopulation of cells that express the NGF Trk-A receptor. The BDNF protein was further transported anterogradely to the dorsal horn of the spinal cord. Li-ESWT suppressed the expression of Trk-A and BDNF in the L6 DRG/spinal cord. Wang et al. found that Li-ESWT significantly increased penis BDNF in a rat erectile dysfunction model by the bilateral crashed cavernous nerve. They suggested that BDNF and PERK/ATF4 pathways could be the specific targets for Li-ESWT [32] to repair the injured nerve tissue. Matsuda et al. showed a therapeutic effect of Li-ESWT evidenced by upregulation of BDNF expression in neural cells at the lesion of a thoracic spinal cord contusion injury, decreased neural tissue damage, and improvement of the locomotor and sensory functions in a rat model [33]. These findings indicate that Li-ESWT provides a neuroprotective effect on the injured nerve tissue through increasing the BDNF expression. However, in the neuroinflammatory condition of CP/CPPS, Li-ESWT lowered the BDNF levels and attenuated inflammation and pain. We suggest that the effects of Li-ESWT are dependent on the tissue conditions, which might induce different molecular changes and induce other biological effects.

It has been demonstrated that inflammation of peripheral tissue significantly increases the expression of COX-2 in DRG neurons [34]. The inflamed tissue and infiltrated inflammatory cells increase prostaglandin secretion, which stimulates sensory neurons through the paracrine effect. The ascending neural pathway for pain signaling is composed of the DRG and spinal cord with the expression of COX-2. Noxious peripheral stimuli-induced synaptic transmission of nociceptive signals from the nociceptor neurons to the superficial dorsal horn of the spinal cord [35]. Our previous study revealed that IL-1β and COX-2 were increased in the inflammatory prostate. Furthermore, this study confirmed the COX-2 overexpression in the L6 DRG and spinal cord, which was downregulated by Li-ESWT. Li-ESWT inhibited the inflammatory molecules in the peripheral organs and central nervous systems.

BOLD fMRI has been used as a tool to monitor the brain’s responses to various noxious stimulations. It provides additional spatial information, which leads to a further understanding of supraspinal pain processing mechanisms [36]. In this study, we found intraprostatic capsaicin injection elicited robust activation in the caudate–putamen, lateral PAG, and thalamus, and brain activation was decreased by Li-ESWT. These findings might provide additional evidence for the central effects of Li-ESWT in improving hyperlocomotion and suppressing of prostatic pain in the rat prostatitis model [9]. Capsaicin as an exogenous ligand for the TRPV1 receptor might exert activation in brain regions of the pain neural circuit, Papez circuit, and habenular system. Previous studies have identified that capsaicin failed to elicit the robust BOLD activity in TRPV1 knockout rats, which was observed in wild-type controls [37]. Another study evaluated the fMRI changes in the spared nerve injury chronic neuropathic pain model to examine the functional changes in the brain associated with the development and maintenance of cold and mechanical hypersensitivity. The extensive functional reorganization in pain-related brain circuitry involves diverse changes in somatosensory as well as cingulate cortices and subcortically within the thalamus and PAG. These changes are reflected in the progression from an acute and adaptive pain state to a chronic, maladaptive neuropathic disease state [38]. Li-ESWT targeted on the prostate decreased the TRPV1 expression in both peripheral and central neural pathways and induced changes in the brain activity in pain-related areas, which findings were coinciding with previous studies and further confirmed that Li-ESWT could modulate the brain pain circuits.

**Figure 7:**
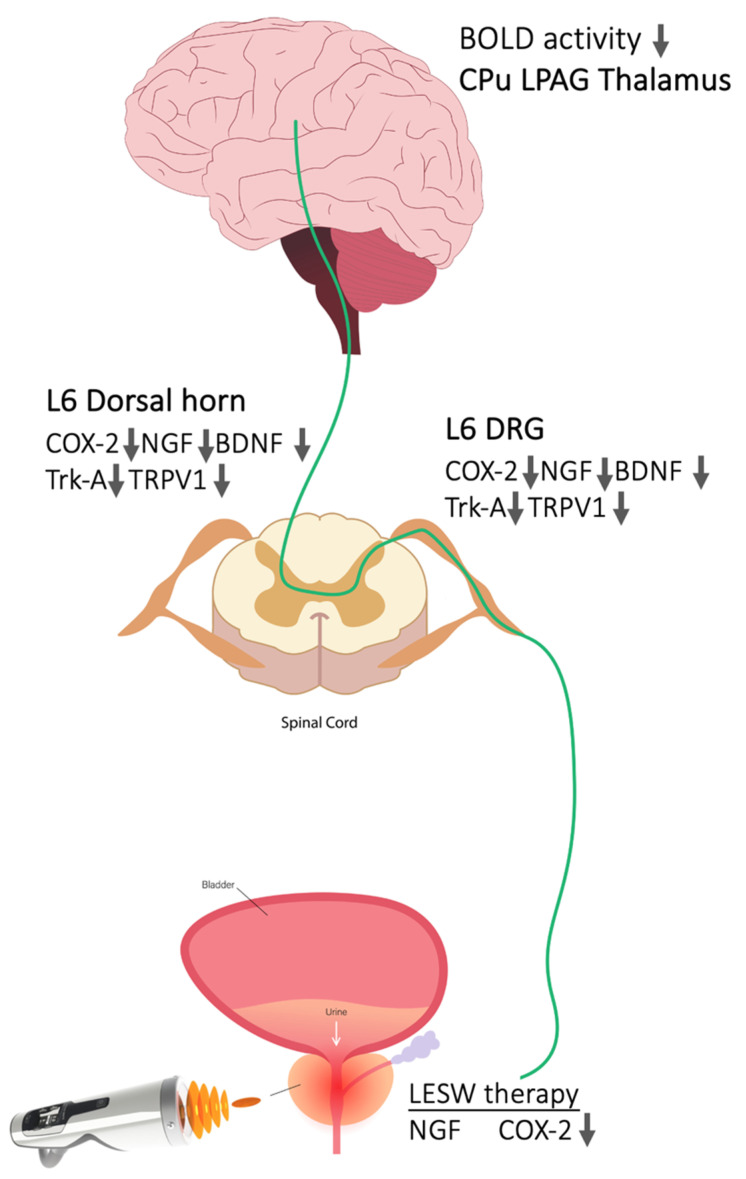
A model illustrating LiESWT treatment on prostate inhibiting the nociceptive molecular and downregulating central sensitization in capsaicin-induced prostatitis in rats. Li-ESWT on prostate decreased the COX-2, NGF expression in the prostate in association with attenuated inflammatory and neurotrophic molecular expression in DRG and its relay neurons at dorsal horn of spinal cord. This anti-nociceptive effect projected through the ascending pathway to the brain.

Immunohistochemistry staining of L6 DRG showed the most robust change in the 200 shocks Li-ESWT group, with a limited effect in the 300 group, but the immunofluorescence staining and Western blot measurements did not mirror these results. For quantification in immunohistochemistry staining, we randomly selected four fields and the positive neurons were counted at 200× magnification. The inconsistent results between IHC and Western blot might come from the random selection visual field in the DRG. The limitation of this study is that although the animal model we used allows us to understand the therapeutic impact of shock wave therapy on the mechanism of pain relief from the prostate to the DRG, spinal cord, and brain, there is still a gap between the expression of these neurogenic prostatitis rat models and the actual CP/CPPS in humans. Thus, the therapeutic effects of shock waves on the modulation of brain circuits that control chronic pain require more evidence and human data to confirm their true value. Nevertheless, we proposed the sequence of central pain processing and suggested that Li-ESWT decreased COX-2 and NGF expression in the prostate and retrogradely attenuated the nociceptive effect at DRG and its relay neurons at the dorsal horn of the spinal cord projected through the ascending pathway to the brain (Figure 7).

## 4. Materials and Methods

### 4.1. Experimental Animals and Study Design

The experiment’s protocol is illustrated in Figure 1. All experimental procedures were performed in male Sprague–Dawley (SD) rats weighing 300–350 g provided by Charles River Technology, BioLASCO Taiwan Co., Ltd., Taiwan. Animals were housed under constant temperature and humidity and 12-h light and dark cycles. All procedures were reviewed and approved by the Institutional Animal Care and Use Committee of Chang Gung Memorial Hospital (IACUC number. 2017122206, approval date: 7 February 2018) and complied with the NIH Guide for Care and Use of Laboratory Animals. Male SD rats were randomized into five groups (*n* = 8 in each group): (1) sham control, with low laparotomy and vehicle injection; (2) prostatitis group, intraprostatic capsaicin injection (CAP); (3) prostatitis rats treated with low-energy extracorporeal shock wave (LESW) at an energy level of 0.12 mJ/mm^2^, 100 impulses (CAP + LESW100); (4) prostatitis rats treated with LESW at 0.12 mJ/mm^2^, 200 impulses (CAP + LESW200); (5) prostatitis rats treated with LESW at 0.12 mJ/mm^2^, 300 impulses (CAP + LESW300). To evaluate brain BOLD signal changes, animals were divided into the following three groups (*n* = 8 in each group): (1) sham control, (2) prostatitis group, and (3) prostatitis rats treated with LESW at an energy level of 0.12 mJ/mm^2^, 200 impulses (CAP + LESW200).

### 4.2. Capsaicin Injection-Induced Prostatitis

Under 2% isoflurane anesthesia, low laparotomy was conducted to explore the prostate for capsaicin injection. Capsaicin was obtained from Sigma-Aldrich (St. Louis, MO, USA). Then, 0.1-mL vehicle (10% alcohol, 10% Tween 80, and 80% saline) with or without capsaicin (10 mM) was directly injected into ventral lobes of the prostate using a 30-gage needle.

### 4.3. Low-Energy Shock Wave Treatments

The skin was closed in layers after intraprostatic capsaicin injection, and the shock wave probe (SD-1, STORZ MEDCAL, Tägerwilen, Switzerland) was gently placed over the skin surface above the prostate area after application of ultrasound transmission gel. Transcutaneous shock wave therapy at different energy flux densities (100, 200, or 300 pulses; 0.12 mJ/mm^2^; 2 Hz) was conducted immediately and 24 h after capsaicin injection [9].

### 4.4. Immunohistochemistry and Immunofluorescent Studies for the L6 Spinal Cord and L6 Dorsal Root Ganglia

Three or seven days after Li-ESWT, the rats were humanely sacrificed using Zoletil 50 (25–50 mg/kg) and Rompun (10–15 mg/kg intramuscularly), followed by transcardiac perfusion with Krebs buffer and retrieval of the L6 DRG/spinal cord. The L6 DRGs were collected and divided into two parts. One part was fixed in 4% paraformaldehyde embedded in paraffin for staining with immunohistochemistry using a commercially available kit (Thermo Scientific UltraVision Quanto Detection system, Fremont, CA, USA). The other part was frozen in liquid nitrogen and preserved for Western blotting. The protocols for immunohistochemistry examinations were described in a previous study [9]. L6 DRG was cut into 5-μm-thick serial sections. After antigen retrieval, endogenous peroxidase activity was blocked with 3% hydrogen peroxide. DRG sections were incubated with primary antibodies, specifically rabbit against COX-2 (1:1000, Cayman Chemical, Ann Arbor, MI, USA), rabbit against brain-derived neurotrophic factor (BDNF) (1:500, Abcam, Cambridge, MA, USA), and rabbit against NGF (1:500, Abcam, Cambridge, MA, USA), and then diluted in antibody diluent solution (Zymed, Thermo Scientific, Carlsbad, CA, USA) for 30 min at room temperature. After being washed in PBS (pH 7.0), sections were incubated in BioGenex Super EnhancerTM Reagent and BioGenex Polymer Horseradish Peroxidase Complex for 20 min in each procedure. Slides were developed with 3,3-diaminobenzidine chromogen (BioGenex DAB substrate) and counterstained with Mayer’s hematoxylin. Slides were then dehydrated and mounted. For quantification, four to six fields were randomly selected, counting at 200× magnification using a 10 × 20 grid in the eyepiece. An AlphaImager 2200 Imaging System (Genetic Technologies, Inc. Miami, FL, USA) was used to calculate COX-2-, NGF-, and BDNF-positive cells in each section.

For immunofluorescent analysis, L6-DRGs were harvested, post-fixed at 4% paraformaldehyde for 4 h, and cryo-protected overnight by passing through a 10% to 30% sucrose gradient. Briefly, DRG frozen sections (25 μm) were incubated with blocking solution containing 10% goat serum in PBST (0.3% Triton X-100 in 0.1 M PBS, pH 7.4) for 1 h at room temperature. The sections were then incubated with primary antibodies, specifically rabbit against NGF (1:200, Abcam, Cambridge, MA, USA), rabbit against BDNF (1:150, Abcam, Cambridge, MA, USA), rabbit against TRPV1/ VR1(1:150, Bioss lnc, Woburn, MA, USA), and rabbit against Trk-A (1:100, Abcam, Cambridge, MA, USA), and were diluted in PBS at 4 °C overnight. After three washes with PBS, the sections were incubated with the secondary antibodies, namely, with Alexa Fluro488-conjugated donkey against rabbit antibody (1:200, Abcam, Cambridge, MA, USA), Alexa Fluro488-conjugated goat against rabbit antibody (1:400, Abcam, Cambridge, MA, USA), or Alexa Fluro594-conjugated goat against rabbit antibody (1:400, Abcam, Cambridge, MA, USA), for 1 h at room temperature. After several washes with PBS, sections were incubated in DAPI (40,60-diamino-2-phenylindole,1:1000; Sigma-Aldrich, MO, USA). Finally, all sections were mounted with a mounting medium (Thermo Fischer Scientific, MA, USA), and representative images were obtained using a Zeiss microscope (Oberkochen, Germany) using the AxioVision software (Version 4.8, Carl Zeiss LLC, Oberkochen, Germany).

### 4.5. Western Blotting

L6–S1 segments of the spinal cord and L6-DRG protein were extracted from grounded frozen tissue for Western blot analysis according to a previous protocol [9]. The samples were homogenized with a protein extraction solution before sonication and purification. The total amount of protein was measured via Bradford protein assay. An aliquot of the extracts equivalent to 30 µg protein was loaded onto 4–20% Mini-PROTEAN TGX Precast Gels (Bio-Rad Laboratories, Hercules, CA, USA), electrophoresed at a constant voltage of 100 V for 1.5 h, and transferred to Hybond-P PVDF Membrane. The membrane was blocked with 5% skim milk powder (Fluka; Sigma-Aldrich, St. Louis, MO, USA) for 60 min and immunoblotted overnight at 4 °C with mouse Anti-Glyceraldehyde-3-Phosphate Dehydrogenase Monoclonal Antibody (as internal loading control) and a primary antibody against COX-2, NGF, BDNF, tropomyosin receptor kinase A (Trk-A) (Abcam, Cambridge, MA, USA), and transient receptor potential vanilloid 1 (TRPV1) (Bioss lnc. Woburn, MA, USA). After being washed, the membrane was incubated with a secondary antibody using a horseradish peroxidase-linked anti-rabbit or anti-mouse immunoglobulin G (diluted concentration 1:5000–1:10,000). The total amount of GAPDH (clone 6C5; 1:5000, EMD Millipore/Chemicon, Burlington, MA, USA) was detected as the internal control. Western blots were visualized by an enhanced chemiluminescence (ECL) detection system (Amersham Biosciences Marlborough, MA, USA). Quantitative analysis was performed using LabWorks™ Image Acquisition and Image Lab software (Bio-Rad Laboratories, Inc. Berkeley, CA, USA).

### 4.6. MR Image Acquisition and Parameters

**Figure 8:**
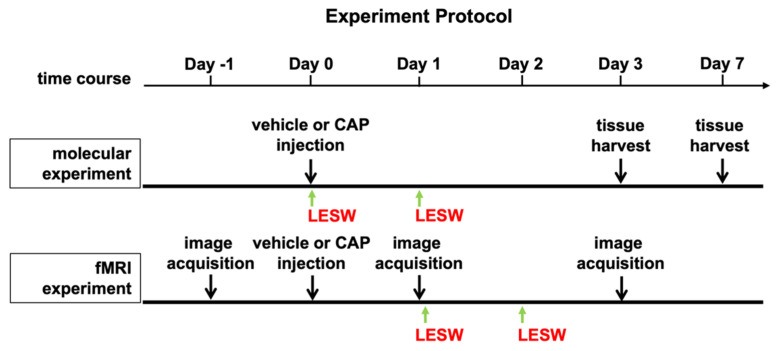
Schematic diagram of experimental protocol. For molecular experiments, the rats received ESWT immediately and 24 h after capsaicin injection. For BOLD fMRI experiments, all the rats received fMRI image acquisition one day before capsaicin injection. One day after intraprostatic capsaicin or vehicle injection, the rats received second fMRI scan. Li-ESWT group received two times of treatment right after and 24 h after second image acquisition. Three days after capsaicin injection the third time fMRI scan was conducted.

The schematic illustration of experiment method is shown in Figure 8. To determine the effect of capsaicin injection on brain activity and reduce the interference of surgical pain on fMRI, the first shock wave therapy was applied 1 day after capsaicin injection and immediately before the first fMRI. Brain activities of rats were systematically evaluated under fMRI scan at identical conditions by administering the intramuscular injection of anesthesia (zoletil, 50 mg/kg). Experimental parameters of MRI are as follows: fMRI analysis was performed on a 9.4-T horizontal-bore animal MR scanning system (Biospec 94/20, Bruker, Ettingen, Germany). The MRI scan imaging system is equipped with a self-shielded magnet (20 cm clear bore and a BGA-12S gradient insert, inner diameter: 12 cm), which offers maximum gradient strength (675 mT/m) for examining the whole rat brain efficiently. Multi-slice turbo rapid acquisition with refocusing echoes (Turbo-RARE) sequence was applied for obtaining the MRI functional images. Specifically, the following parameters were applied: field of view (FOV) = 35.0 × 17.5 mm; matrix size = 256 × 128; spatial resolution = 137 × 137 μm; slice thickness = 0.5 mm; effective echo time (TE) = 30.0 ms; echo time = 10 ms; repetition time (TR)= 3000 ms; rare factor = 8; refocusing flip angle = 180 deg.; number of averages = 2; number of repetitions (NR) = 1; total acquisition time = 1 min 36 s. T2 -weighted 2D RARE axial anatomical reference imaging was performed on 15 adjacent slices from a restricted area of the brain that included the PAG using Turbo-RARE sequence acquisition with the following parameters: field of view (FOV) = 25.0 × 25.0 mm; matrix size = 256 × 256; spatial resolution = 98 × 98 μm; slice thickness = 1.0 mm; effective echo time (TE) = 30 ms; echo time = 10 ms; repetition time (TR) = 3000 ms; rare factor = 8; refocusing flip angle = 180 deg.; number of averages = 5; number of repetitions (NR) = 1; total acquisition time = 8 min. By using identical spatial dimensions in the T2-weighted axial reference imaging, we acquired the blood oxygenation level-dependent (BOLD) contrast-sensitive for functional images through the echo-planar imaging (EPI) sequence with the following parameters: field of view(FOV) = 25.0 × 25.0 mm; matrix size = 96 × 96; spatial resolution = 260 × 260 μm; slice thickness = 1.0 mm; effective spectral bandwidth = 250,000 Hz; echo time (TE) = 20.0 ms; repetition time (TR) = 2000 ms; segment = 1; number of averages = 1; total acquisition time = 4 min. One hundred twenty EPI volumes were acquired for each run. Rats were in resting state during all the imaging sessions.

### 4.7. Post-Processing of Images Data Analysis

Post fMRI analysis, all the obtained images were processed by using conventional software/procedures. Briefly, Statistical Parametric Mapping (SPM8, Wellcome Department of Cognitive Neurology, London, UK) software was used for spatial smoothening (FWHM = 1 mm), white matter/ventricle signals, regressions of motion parameters, and band-pass filtering (0.002–0.1 Hz). Notably, excessive motion (max within scan displacement >0.5 mm) in the resting state-fMRI (rs-fMRI) was discarded. Based on the literature protocol, we identified the single intensity in the PAG region of the rat brain and analyzed the data by using imageJ 1.51 [39].

Further voxel-based analysis was performed in order to determine the accurate normal fluctuation of BOLD signal in the rat brain. Each independent voxel was averaged and substracted with the 5% BOLD baseline threshold [40]. Thus, final region of interest (ROI) analyses of the whole brain images representing the average of all subjects include caudate putamen (CPu), lateral PAG (LPAG), and thalamus (Thus). From the ROI data of each subject, we can clearly estimate/determine/observe the BOLD changes as well as number of activated voxels in thalamus. Subsequently, we also compare the effects of capsaicin and LESW therapy over time.

### 4.8. Statistical Analysis

Quantitative data are expressed as mean ± standard error of the mean. Statistical analyses were conducted using two-way ANOVA, with the Bonferroni post hoc test where applicable, with *p* < 0.05 considered significant. Statistical analyses were conducted using GraphPad Prism v.7 (GraphPad Software, Inc., La Jolla, CA, USA) for biostatistics.

## 5. Conclusions

Intraprostatic capsaicin injection activates COX-2, NGF, and related neurotrophin expression in the L6 DRG/spinal cord. Li-ESWT modulates neurotrophin expression at both the peripheral and central nervous systems and pain circuits of the brain in a dose-dependent manner. This finding suggests a potential clinical benefit of Li-ESWT in the modulation of the chronic pain process through peripheral nerve systems as well as central nerve systems.

## Figures and Tables

**Table 1:** Western blotting expression of inflammatory and neurotrophin molecular in capsaicin and Li-ESWT group relative to sham control group on days 3 and 7 in L6-DRG neurons (*n* = 10 for each group). * *p* < 0.05; # *p* < 0.01; † *p* < 0.001.

		Adjusted *p*-Value (Bonferroni’s Multiple Comparisons Test)								
		Sham Control	Capsaicin (Cap.)	+Cap. LESW100	+Cap. LESW200	+Cap. LESW300	Cap.vs. Sham	+Cap. LESW100 vs. Cap.	+Cap. LESW200 vs. Cap.	+Cap. LESW300 vs. Cap.
**Day 3**	COX-2	1.00 ± 0.00	2.13 ± 0.24	1.50 ± 0.19	1.16 ± 0.22	0.97 ± 0.17	† <0.001	*NS.*	# <0.01	† <0.001
	NGF	1.00 ± 0.00	1.38 ± 0.06	0.96 ± 0.11	0.86 ± 0.06	0.86 ± 0.06	# <0.01	† <0.001	† <0.001	† <0.001
	Trk-A	1.00 ± 0.00	1.56 ± 0.21	1.01 ± 0.11	0.83 ± 0.12	0.69 ± 0.095	* <0.05	* <0.05	# <0.01	† <0.001
	TRPV1	1.00 ± 0.00	1.60 ± 0.17	1.14 ± 0.09	1.01 ± 0.08	0.91 ± 0.13	# <0.01	*NS.*	* <0.05	† <0.001
	BDNF	1.00 ± 0.00	1.47 ± 0.18	1.02 ± 0.09	0.82 ± 0.08	0.68 ± 0.10	* <0.05	* <0.05	† <0.001	† <0.001
**Day 7**	COX-2	1.00 ± 0.00	2.06 ± 0.24	1.68 ± 0.26	0.93 ± 0.13	1.14 ± 0.17	<0.01	*NS.*	<0.001	<0.01
	NGF	1.00 ± 0.00	1.43 ± 0.14	1.19 ± 0.07	1.01 ± 0.07	0.95 ± 0.08	<0.01	*NS.*	<0.05	<0.01
	Trk-A	1.00 ± 0.00	1.68 ± 0.27	0.97 ± 0.07	0.84 ± 0.06	0.91 ± 0.11	<0.05	<0.01	<0.001	<0.01
	TRPV1	1.00 ± 0.00	1.51 ± 0.18	0.98 ± 0.06	0.97 ± 0.12	1.04 ± 0.11	<0.05	<0.05	<0.05	<0.05
	BDNF	1.00 ± 0.00	1.44 ± 0.13	1.21 ± 0.13	0.87 ± 0.10	0.73 ± 0.11	<0.05	*NS.*	<0.01	<0.001

The table show general characteristics of the experimental animals. Data presented as the means ± SEM of 10 rat/group.

**Table 2:** Western blotting expression of inflammatory and neurotrophin molecular in capsaicin and Li-ESWT group relative to sham control group on days 3 and 7 in dorsal horn of L6 spinal cord (*n =* 10 for each group). * *p* < 0.05; # *p* < 0.01; † *p* < 0.001.

		Adjusted *p*-Value (Bonferroni’s Multiple Comparisons Test)								
		Sham Control	Capsaicin (Cap.)	+Cap. LESW100	+Cap. LESW200	+Cap. LESW300	Cap.vs. Sham	+Cap. LESW100 vs. Cap.	+Cap. LESW200 vs. Cap.	+Cap. LESW300 vs. Cap.
**Day 3**	COX-2	1.00 ± 0.00	2.20 ± 0.29	1.42 ± 0.25	1.02 ± 0.14	0.95 ± 0.15	† <0.001	*NS.*	# <0.01	† <0.001
	NGF	1.00 ± 0.00	1.33 ± 0.095	1.01 ± 0.09	0.76 ± 0.05	0.83 ± 0.06	* <0.05	* <0.05	† <0.001	† <0.001
	Trk-A	1.00 ± 0.00	1.70 ± 0.12	1.34 ± 0.11	0.87 ± 0.23	0.90 ± 0.12	# <0.01	*NS.*	† <0.001	† <0.001
	TRPV1	1.00 ± 0.00	1.63 ± 0.17	1.42 ± 0.18	0.87 ± 0.10	0.97 ± 0.16	* <0.05	*NS.*	# <0.01	* <0.05
	BDNF	1.00 ± 0.00	1.99 ± 0.19	1.60 ± 0.32	0.94 ± 0.12	0.96 ± 0.18	* <0.05	*NS*.	* <0.05	# <0.01
**Day 7**	COX-2	1.00 ± 0.00	2.25 ± 0.35	1.13 ± 0.18	0.84 ± 0.05	1.17 ± 0.30	<0.01	<0.01	<0.001	<0.05
	NGF	1.00 ± 0.00	2.10 ± 0.43	1.09 ± 0.10	1.00 ± 0.12	0.91 ± 0.16	<0.01	<0.05	<0.01	<0.01
	Trk-A	1.00 ± 0.00	2.58 ± 0.70	1.04 ± 0.054	1.00 ± 0.10	1.52 ± 0.43	<0.05	<0.05	<0.05	*NS.*
	TRPV1	1.00 ± 0.00	1.91 ± 0.29	0.84 ± 0.081	0.85 ± 0.06	1.21 ± 0.23	<0.01	<0.001	<0.001	*NS.*
	BDNF	1.00 ± 0.00	1.68 ± 0.23	1.04 ± 0.099	0.91 ± 0.08	0.91 ± 0.14	<0.01	<0.01	<0.001	<0.01

Data presented as the means ± SEM of 10 rats/group.

**Table 3:** Capsaicin intraprostatic injection induced fMRI bold signal changes in brain. Regions of interest including thalamus, lateral periaqueductal grey and caudate putamen of rats. Data presented as the average number of active voxels in ROI. Data shown in means ± SEM. *n* = 8 per group. * *p* < 0.05; # *p* < 0.01; † *p* < 0.001.

	Average Number of Active Voxels			Significant (Tukey’s Multiple Comparisons Test)		
	Sham Control	Capsaicin (CAP)	CAP + LESW200	Sham vs. Cap	CAP vs. CAP + LESW200	CAP + LESW200 vs. Sham
**Thalamus; Th**						
pre-injection	62.25 ± 46.29	66.08 ± 43.93	94.00 ± 41.59	*NS*	*NS*	*NS*
postcapsaicininjection, day1	209.20 ± 70.59	259.60 ± 82.05	410.83 ± 38.06	*NS*	*NSNS*	
postcapsaicininjection, day3	217.71 ± 102.77	459.830 ± 89.98	83.50 ± 43.04	*	#	*NS*
**Lateral PAG; LPAG**						
pre-injection	50.25 ± 20.77	39.17 ± 12.61	44.40 ± 11.90	*NS*	*NS*	*NS*
postcapsaicininjection, day1	62.60 ± 13.94	93.00 ± 16.24	92.60 ± 10.50	*NS*	*NS*	*NS*
postcapsaicininjection, day3	28.8 ± 7.61	54.50 ± 6.54	12.17 ± 3.68	*NS*	*	*NS*
**caudate putamen; CPu**						
pre-injection	71.00 ± 48.61	49.20 ± 35.03	69.88 ± 25.44	*NS*	*NS*	*NS*
Postcapsaicininjection, day1	193.60 ± 26.34	302.75 ± 23.35	455.00 ± 25.86	*NS*	*NS*	*
postcapsaicininjection, day3	209.71 ± 98.66	387.43 ± 106.39	49.00 ± 20.15	*NS*	†	*NS*

## Data Availability

The data presented in this study are available on request from the corresponding author.
